# Developing theory-informed implementation strategies to embed a suicide safety planning intervention app into a psychiatric emergency department: co-design study using the Behaviour Change Wheel

**DOI:** 10.1192/bjo.2025.10824

**Published:** 2025-09-12

**Authors:** Hwayeon Danielle Shin, Gillian Strudwick, John Torous, Keri Durocher, Juveria Zaheer

**Affiliations:** Institute of Health Policy, Management and Evaluation, University of Toronto, Toronto, Ontario, Canada; Centre for Addiction and Mental Health, Toronto, Ontario, Canada; Psychiatry, Beth Israel Deaconess Medical Center, Boston, Massachusetts, USA; Arthur Labatt Family School of Nursing, Western University, London, Ontario, Canada; Department of Psychiatry, Temerty Faculty of Medicine, University of Toronto, Toronto, Ontario, Canada

**Keywords:** Implementation, suicide prevention, co-design, health technology, nursing informatics

## Abstract

**Background:**

Safety planning is a commonly used, evidence-based intervention for suicide prevention. There is a need for continuous engagement with safety plans post-discharge, and the improvement of safety plan portability has been discussed within our mental health organisation. This has led to the development of an app, called the Hope App. This study aims to implement this app into routine practice in a Canadian psychiatric emergency department.

**Aims:**

We aimed to describe a collaborative, theoretically driven approach to co-design implementation strategies to elicit behaviour change among emergency department clinicians; co-develop a set of tailored, theory-informed, multifaceted implementation strategies for embedding an app into a psychiatric emergency department; and describe engagement evaluation received by the co-design team.

**Method:**

Co-design approaches and the Behaviour Change Wheel were used to develop implementation strategies with clinicians, patients and care partners. The co-design team consisted of 12 members, and we held four design sessions. Design sessions were iterative in nature and organised such that the findings of each session fed into the next session.

**Results:**

We identified 11 implementation strategies encompassing different combinations of intervention functions and behaviour change techniques, targeting barriers and leveraging facilitators identified in our previous work.

**Conclusions:**

The tailored implementation strategies developed in this study have the potential to fill existing gaps in integrating digital technology. A key strength of this study is its use of behaviour change theories and a collaborative approach. The strategies are designed to align with the needs and preferences of clinicians, patients and care partners.

The emergency department is a frequent location to seek help for suicide-related thoughts and behaviours. One important aspect of suicide prevention is a safety planning intervention (SPI), which is an evidence-based intervention that has shown to reduce the risk of suicidal behaviour.^1^ A safety plan is personalised for each patient, and at our local psychiatric emergency department in Toronto, Canada, a paper-based SPI is a standard discharge practice. An SPI is provided to individuals who are stable enough to go home after a thorough mental health assessment and appropriate referrals, and it is anticipated that individuals engage with their SPIs for symptoms management, including, but not limited to, coping strategies, maintaining a safe environment and contacting their safety net. Recognising the need for continuous engagement with SPIs and the subsequent need for improved portability, our organisation has co-developed an SPI app with clinicians and patient partners, called the Hope App (version 1.2.2; Centre for Addiction and Mental Health, Toronto, Ontario, Canada). This is free to the public, and available for both android and iOS devices.^2^

Developing innovations with end-users has become a common practice. However, co-creating changes in the clinical workflows to allow seamless integration of innovations remains inadequate, as many abandoned digital tools testify to this reality.^3^ Moreover, digital health technology implementations often lacks theoretical guidance.^4,5^ This gap in the literature underscores the need to consider multiple layers of health system factors and to conceptualise implementation as a behaviour change process, guided by relevant theories, models or frameworks. Introducing new innovations into practice requires changing the old way of doing things, which requires changing clinicians’ behaviour. It is widely recognised that implementation efforts are most effective when guided by behaviour change theory.^6^

Traditionally, patients and their caregivers have not been involved in developing new care approaches in the emergency department or mental healthcare. High-acuity settings like the emergency department may also seem difficult setting to use a collaborative approach. However, SPI is collaborative in nature, meaning it should ideally be co-developed by patients and their providers. Increasingly, the literature suggests involving caregivers during mental health crises,^7,8^ and these situations often require an SPI. Caregivers of individuals at risk of suicide want recognition for the valuable insights they provide, and for their ability to have a positive impact on the interactions between the patient and clinician.^9^ Their involvement can extend to the research context and bring significant impact.^10,11^ People with lived experience of suicide-related thoughts and behaviours also play a critical role in the design^12^ and delivery^13^ of suicide prevention programmes and interventions. As such, the way we generate evidence to inform practice change is an opportunity to include key people who will be affected by research findings.^14^

Integrated knowledge translation (IKT) is historically a Canadian terminology for a collaborative model of research, where researchers work with knowledge users (e.g. managers, clinicians) from the initial identification of the problem to co-design and evaluate the solution to implementation challenges. Before the current study, clinicians working in the psychiatric emergency department had identified barriers and facilitators of using the Hope App to support SPI.^15^ In this current study, we had the following aims:

- To describe a collaborative, theoretically driven approach to co-designing implementation strategies to elicit behaviour change among emergency department clinicians.
- To co-develop a set of tailored, theory-informed, multifaceted implementation strategies for embedding an SPI app into a psychiatric emergency department.
- To describe engagement evaluation received by the co-design team, highlighting the impact of collaboration.

## Method

### Design

This study was a part of a larger project, and the full protocol is published elsewhere.^16^ This paper presents the results of the final phase. We used an IKT approach for this co-design study, where clinicians, who use research findings, and patients and caregivers (i.e. families and friends), who are affected by research findings, are partnered to ensure research findings are applicable in the study’s implementation context. Another important assumption of IKT is that both researchers and partners are experts, and shared decision-making is anticipated. The GRIPP2 reporting guide^17^ for patient and public engagement was used to prepare this manuscript. The Behaviour Change Wheel (BCW) served as the primary theoretical foundation for designing implementation strategies.^6^ At the core of the BCW, the Capability, Opportunity, Motivation – Behaviour (COM-B) suggests an important assumption about human behaviour, namely that capability, opportunity and motivation interact in complex ways to shape behaviour. This model highlights the idea that for a behaviour to occur, individuals must have the necessary capability (skills and knowledge), the opportunity (external physical and social factors or resources) and the motivation (autonomic and reflective) to engage in that behaviour. Absence of any of these necessary COM-B determinants that are specific to the desired behaviour may not lead to successful behaviour change. The COM-B is then connected to nine intervention functions, which represent broad categories of actions aimed at influencing the COM-B components. Additionally, the Behaviour Change Technique Taxonomy version 1 (BCTTv1) complements the COM-B by providing a comprehensive list of specific behaviour change techniques (BCTs) that can be used within each intervention function. Together, these elements create a robust framework that supports the development of targeted implementation strategies designed to promote behaviour change effectively.

### Setting

The psychiatric emergency department is the implementation context for this study and is located in the Greater Toronto Area, Ontario, Canada. Each month, the emergency department provides care to an average of 1300–1500 adult patients. The emergency department was chosen to participate in the study because there had been no organised efforts to integrate the app into the routine clinical workflow. Given the unique nature of the setting, it was clear that tailored and multifaceted implementation efforts were necessary, which was also substantiated by the previous qualitative work.^15^ All design sessions were held virtually, using the organisation-approved video platform, Webex.

### Participants

Three types of designers were recruited for this study: (a) emergency department clinicians, (b) emergency department patients (i.e. lived experience experts who have used the emergency department for their suicide-related thoughts and behaviours) and (c) care partners (i.e. individuals who looked after their families or friends living with suicide-related thoughts and behaviours). Emergency department clinicians who expressed interest in the participation in this co-design study during the previous qualitative study were contacted and those who consented joined the team. Additional emergency department clinicians were recruited through email communication and snowball sampling. Emergency department patients and care partners were recruited by leveraging the established partnerships of the internal organisation. All participants completed written consent via REDCap, following our organisation’s standards, all of which were witnessed and formally recorded.

### Co-design sessions to operationalise BCW and APEASE criteria

There were four co-design sessions, and they were planned so that the findings of each session fed into the next session. Each participant evaluated their engagement experience at the end of each session, using a survey adapted from the Public and Patient Engagement Evaluation Tool (PPEET).^18^ The PPEET evaluates on communication and supports for participation, experience with sharing ideas, and perceived impact and influence of engagement. In the following weeks of each session, the co-design team received a summary of session meeting notes. At the start of each design session, they were provided with the summary of the previous session. The details, goals and activities of each design session are described below.

#### Session 1

We spent time to build rapport, and the main goal was to generate insights on what change is needed and feasible to integrate the SPI app into the normal workflow. Ideas were sought for when (i.e. timing within discharge), who (e.g. psychiatrists, nurses or both) and how clinicians should deliver the app. Also, we spent this time to understand the implementation in behavioural terms. We defined the target behaviour change that emergency department clinicians needed to support the use of the SPI app, downloaded on patients’ own devices before discharging patients to home. We also identified additional actions of clinicians, such as providing instructions and technical support associated with the use of the app. The co-designers were presented with the previous findings of behaviour determinants of the SPI app use in the emergency department.

#### Session 2

Barriers and facilitators have been previously identified and mapped onto the COM-B.^15^ Then, these implementation determinants were linked to nine intervention functions, the types of implementation strategies that we need to consider for a successful behaviour change.^19,20^ These functions also map to the BCTTv1, a series of 93 individual BCTs grouped into 16 categories to further specify the ‘active ingredients’ and also the smallest unit that make up implementation strategies to change behaviour.^21^ The selection of BCTs have been guided by the theories of techniques tool, an open-access resource that helps researchers to prioritise BCTs that have shown a strong link with addressing COM-B targets.^22,23^ The prematched implementation strategies that encompass different combinations of intervention functions and BCTs have been presented to the co-design team. During this session, the design team engaged in discussion guided by the APEASE criteria. The APEASE criteria considers whether the suggested implementation strategies are Affordable, Practical, Effective and cost-Effective, Acceptable, Safe and free from unintended negative consequences, and Equitable.^19,20^

#### Session 3

Session 2 findings were organised into implementation strategies and corresponding BCTs. This resulted in 11 implementation strategies, and they were presented to the co-design team, one by one. The implementation strategies and their contents were described following the implementation strategies reporting criteria by Proctor et al, which covered what, who, how, where, when, how much and why (justifications) and intended outcome(s).^24^ The co-design team evaluated 11 strategies and open discussion generated further recommendations. We used the APEASE criteria^19,20^ again to guide our discussion, and this time we used polling to facilitate our evaluation. Co-designers rated each implementation strategy following the APEASE criteria.

#### Session 4

APEASE polling results as well as refined contents for each implementation strategies were presented to the team. The primary author presented 11 implementation strategies and highlighted the incorporated suggestions generated from session 3. We used this meeting to validate what we have developed as a team and generated final recommendations. Moreover, implementation strategies that require material production, such as slide decks and brochures, were briefly presented and shared with the team after the meeting.

### Ethics

The authors assert that all procedures contributing to this work comply with the ethical standards of the relevant national and institutional committees on human experimentation and with the Helsinki Declaration of 1975, as revised in 2013. All procedures involving human subjects/patients were approved by the Research Ethics Board at the Centre for Addiction and Mental Health (approval number 2023-078). To encourage the sharing of ideas, ground rules were introduced during the initial session and reiterated at the beginning of each design session. Another strategy used to enable a comfortable environment was ensuring a higher proportion of patients and caregivers versus clinicians in each meeting.

### Data collection and analysis

We collected co-designers’ characteristics, including their self-rated digital literacy, using the electronic health literacy scale (eHEALS),^25^ and they were analysed with descriptive statistics. Descriptive statistics and frequency counts for the APEASE criteria were also generated for each implementation strategy. APEASE scores were converted to percentages to facilitate comparisons within and across APEASE criteria and recommendations. Each criterion was treated as a dichotomous variable (Yes 1, No 0, Uncertain 0). APEASE criteria scoring 80% or higher were considered ‘good’, 60–79% ‘moderate’, and below 60% ‘poor’.^26^ All design sessions were audio-recorded and transcribed with the built-in transcription function. There was a note-taker in each session, taking notes on team dynamics, salient topics of discussion as well as the chat conversations. The PPEET was evaluated on a 1–5 Likert scale, where 1 represents ‘strongly disagree’ and 5 represents ‘strongly agree.’ Each score was analysed using mean and standard deviation, and any narrative feedback was shared during the next session with the team. The team’s suggestions were incorporated into the next session when possible. The primary author re-listened to the meeting, analysed the transcripts, identified themes related to implementation strategies within the data and interpreted the findings ahead of the next meeting. These insights were used to inform both the content of the subsequent meeting and the development and refinement of the implementation strategies. The co-design meetings served as a validation point, as each meeting started with a recap of the previous session for member-checking purposes. To ensure alignment of each implementation strategy and its corresponding BCTs, two researchers (H.D.S., K.D.) reviewed all implementation strategies and their content for theoretical alignment.

## Results

**Fig. 1:**
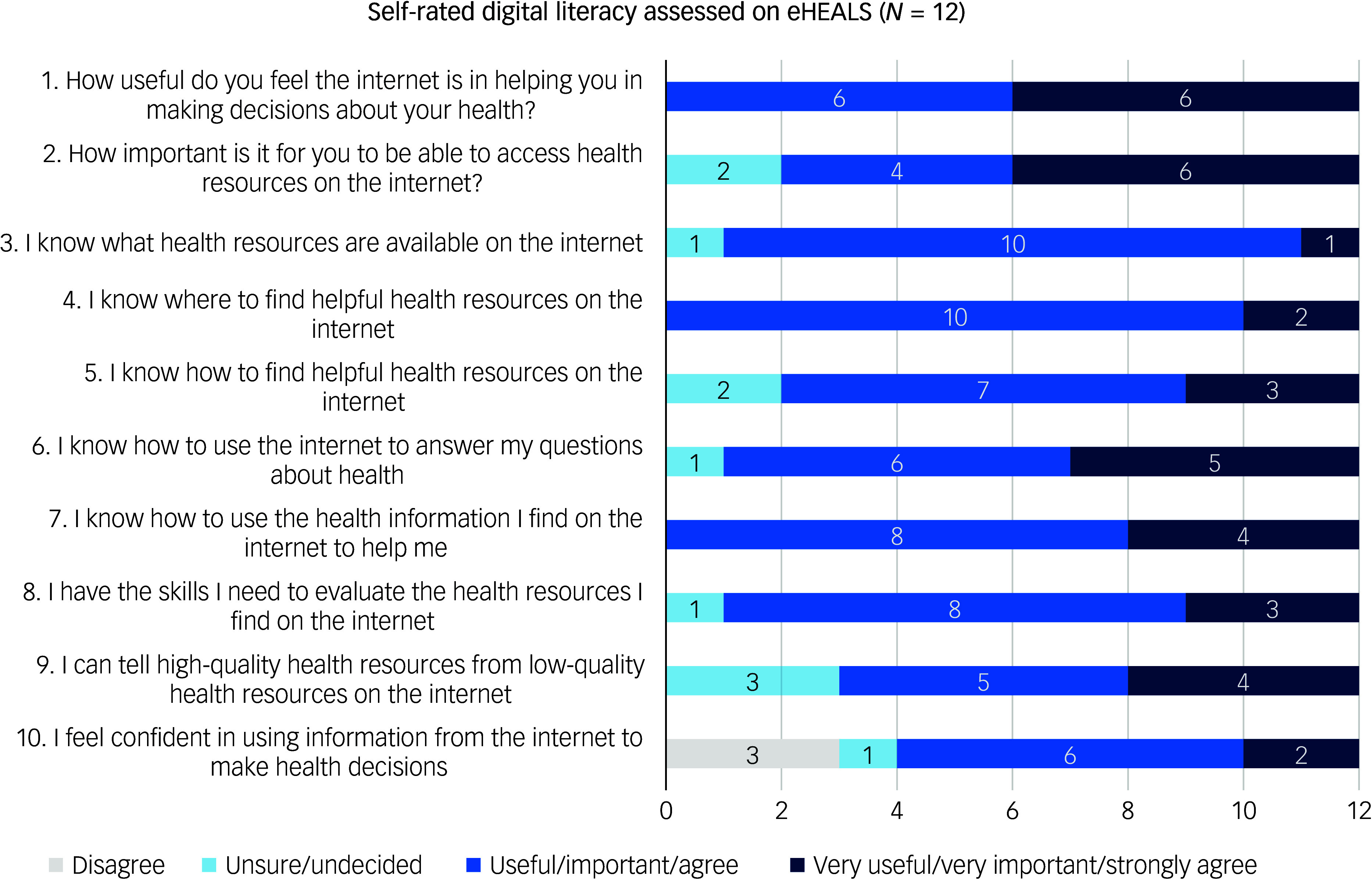
Self-rated digital literacy of the co-design team members. eHEALS, electronic health literacy scale.

Our co-design team consisted of 12 members, including three psychiatric nurses, two psychiatrists and one social worker, all of whom were currently practicing in the psychiatric emergency department. We also included three individuals with lived experience of accessing care in the psychiatric emergency department in the past related to suicide-related thoughts and behaviours, and three individuals who support family or friends living with suicide-related thoughts and behaviours. All members of the team self-reported good levels of digital literacy on eHEALS (Fig. 1). Table 1 presents characteristics of the co-design team. There were six meetings in total across four design sessions. Not all members of the team participated in all four design sessions because of scheduling challenges, but three members (clinician, patient, caregiver) participated in all four sessions, five participated in three sessions and four participated in either two or one session. Overall, the team had a good experience, as noted in the PPEET. Mean scores for each session were as follows: session 1, 4.5; session 2, 4.6; session 3, 4.8; session 4, 4.7. The full results of PPEET are presented in Supplementary File 1 available at https://doi.org/10.1192/bjo.2025.10824.

**Table 1:** Characteristics of co-design team members

Characteristics (*N* = 12)	*n*	%
Clinician	6	50
Years of experience in the current professional role^^a^^		
≤5	1	
6–10	2	
11–20	1	
>20	1	
Current working hours^^a^^		
Full time	4	
Part time	1	
Years of experience working in the current emergency department^^a^^		
≤5	3	
6–10	1	
11–20	1	
Patient	3	25
Family/friend	3	25
English is first language	8	67
Place of residence		
Urban	10	83
Suburban	2	17
Gender		
Women	10	83
Men	2	17
Age, years		
<30	3	25
30–39	4	33
40–49	2	17
50–59	3	25
Race^^b^^		
White	6	50
Racialised (non-exhaustive examples are shown below)	6	50
Black	1	
East or South Asian	2	
Middle Eastern	1	

a Missing value (*n* = 1).

b Non-mutually exclusive category.

The team designed 11 implementation strategies encompassing different combinations of intervention functions and BCTs. Supplementary File 2 provides details including selected BCTs, justifications and the COM-B targets. The subsections below describe the five most significant implementation strategies and Table 2 provides more details on these five strategies, descriptions of BCTs and APEASE rating results. The remaining six strategies can be found in Supplementary File 3.

**Table 2:** APEASE rating from Session 3 and final recommendations from Session 4

Intervention functions	Implementation strategies and description of BCTs	Modes of delivery	**Good**: ≥ 80% agreement **Moderate**: 60–79% agreement **Poor**: <60% agreement						Final recommendations or comments	Relevant quotes
			Affordability	Practicality	Effectiveness	Acceptability	Safety/Side-effects	Equity		
1. Education + training + persuasion	- Include contents of the app in the emergency department new staff orientation (in-person) as part of the suicide prevention module - Content for inclusion: ○ What it is, how it was created, who it is for, why it was created, empirical use cases of an app in emergency department related to safety planning and suicide prevention, demonstrations on how to download, introduce, use, and navigate the app. ○ User’s approvals and testimonials for the app - *Ad-hoc* staff meeting about the app for additional informational support maybe necessary - Actors: nurse educator, advance practice nurse, educators for physicians and social workers - Frequency: align with discipline specific education days (e.g. every month for new nursing staff orientation)	Power point slide deck	Yes: 6 No: 0 Unsure: 1 86%	Yes: 6 No: 0 Unsure: 1 86%	Yes: 7 No: 0 Unsure: 0 100%	Yes: 6 No: 0 Unsure: 1 86%	Yes: 6 No: 0 Unsure: 1 86%	Yes: 7 No: 0 Unsure: 0 100%	- Other contents for consideration: Evaluation of the app using the American Psychiatric Association (APA) framework, download numbers - Include the educational material into the existing training sessions	‘I think this looks good. I mean, if you’re going to integrate something, it really starts with education, and I do like that you’ve incorporated the user testimonials because I think that goes a long way to help him with buy in.’ (Patient) ‘If we can actually use, like, download numbers because I know when I was like doing the presentation of the Hope App, a lot of people like to see, like, so I did the actually the presenting on the numbers and I think that was a huge aspect.’ (Patient) ‘That’s a great idea to integrate it into an existing module.’ (Friend of patient)
2. Incentivisation 1	- Inform and provide certificate of the module (described above) completion	- Online or paper certificate	Yes: 4 No: 1 Unsure: 2 57%	Yes: 4 No: 1 Unsure: 2 57%	Yes: 3 No: 2 Unsure: 2 43%	Yes: 5 No: 0 Unsure: 2 71%	Yes: 6 No: 0 Unsure: 1 86%	Yes: 7 No: 0 Unsure: 0 100%	- Mindful of different degree of motivation associated with certificate - Mostly, recognition is well-received by clinicians - Consider other forms of incentivization, including pin/badge	‘A possible downside of the certification is that it adds to the burden of mandatory modules that clinicians need to complete.’ (Clinician) ‘I am definitely motivated to complete the course, get the certificate, and kind of it’s a little bit of a checkbox of something else I’ve accomplished like for my professional development, but I don’t know how true that is.’ (Patient) ‘I think it’ll also depend on the clinician, but I think having something formalized, and provided to staff training and some kind of recognition that it was done, I think for the most part people respond positive to positively to that and I think it’s something we can try out and if it’s not like if it’s, if it’s not great, then we can, we can stop with like the certificate, but I think the module is still important and would be helpful for our clinicians.’ (Clinician)
3. Education + training + enablement	- During the emergency department morning huddle, dedicate a portion of time for all staff to try a grounding technique or box breathing together, which is one of the contents of the app - Actors: need to find someone capable of leading group activities, preferably non-emergency department staff because of existing workload - Target audience: All emergency department staff on shift - Frequency: every morning for six weeks to cover all staff	- In-person and own telephones	Yes: 6 No: 0 Unsure: 1 86%	Yes: 6 No: 0 Unsure: 1 86%	Yes: 7 No: 0 Unsure: 0 100%	Yes: 6 No: 0 Unsure: 0 100%^a^	Yes: 7 No: 0 Unsure: 0 100%	Yes: 6 No: 0 Unsure: 1 86%	- Need to keep it brief - Explore working with internal team or volunteers (leverage existing human resources) instead of asking emergency department staff to lead the activity	‘This is a good idea, emergency department can be very busy, but this is the best we can do. Huddle is the only time where people have, they can pay attention to or most of the emergency department staff can pay attention.’ (Clinician) ‘I can’t imagine, to be honest, how busy it must get in emergency department, but it’s definitely possible to make these kind of interactivities brief, small doses of it, and diversifying each time a huddle is done, it can just add resources, to, you know.’ (Patient)
4. Coercion + environmental restructuring	- Build in electronic health record documentation for the delivery of SPI and its mode of delivery - Introduce checkboxes in the documentation form to indicate completion of SPI, and to specify the mode of delivery (i.e. app versus paper) of SPI - Actors: advance practice nurse, emergency department leadership, IT team - Frequency: one time change	- EHR form	Yes: 7 No: 0 Unsure: 0 100%	Yes: 7 No: 0 Unsure: 0 100%	Yes: 7 No: 0 Unsure: 0 100%	Yes: 7 No: 0 Unsure: 0 100%	Yes: 7 No: 0 Unsure: 0 100%	Yes: 7 No: 0 Unsure: 0 100%	- Check boxes seem sufficient but consider providing optional free-text area for narrative data (e.g. why it was not delivered)	‘I think the checkboxes at the end is enough.’ (Clinician) ‘Once that is embedded, it will be helpful to know who of the clinical team members would be responsible for actually implementing it just so that it’s clear.’ (Clinician) ‘One of the suggestions I had was under the box where it says safety plan delivered, if there’s a section where you can type in what barriers you face if you weren’t able to deliver it, so like maybe the patient refused or there was enough time or whatever, but I’m just thinking that might be an interesting place to collect some information on why couldn’t it happen.’ (Friend of patient) ‘I think that’s a good idea because if we ever have to like do an evaluation, we can, we can pull those discrete fields but also look at what the qualitative comments were as well.’ (Clinician)
5. Persuasion	- Collect data to evaluate the impact of safety planning and the app - Actors: advance practice nurse, emergency department leadership, IT team - Frequency: evaluation as needed	- EHR enabled data collection - In-person staff rounds for sharing outcomes data	Yes: 4 No: 0 Unsure: 3 57%	Yes: 5 No: 0 Unsure: 2 71%	Yes: 6 No: 0 Unsure: 1 86%	Yes: 5 No: 0 Unsure: 2 71%	Yes: 6 No: 0 Unsure: 1 86%	Yes: 5 No: 0 Unsure: 2 71%	- Need to start from minimal amount of data collection for evaluation - Need to be clear that data collection is from the EHR not from the app, as this can hinder trust - Consider requesting report to data analytics team once EHR form has been built - Consider conducting larger scale user experience evaluation in the future	‘We could request a report from our reporting and analytic analytics team for the data so they can just you know pull the number of completed from the charts and send that to us on either monthly basis or a regular basis.’ (Clinician) ‘This strikes me as being a really good idea. I don’t know the back end or how it would work, but this strikes me as something that would be good to do.’ (Patient) ‘Idea of collecting numbers, but how do we do it without affecting privacy and because we need patients to be able to trust the app, to be able to use it.’ (Patient)

APEASE, Affordable, Practical, Effective and cost-Effective, Acceptable, Safe and free from unintended negative consequences, and Equitable; BCT, behavioural change technique; EHR, electronic health record; SPI, safety planning intervention.

a. Missing value (*n* = 1).

### Education, training and persuasion

One barrier prioritised by the co-design team was the limited awareness and the subsequent lack of buy-in for the SPI app among the emergency department clinicians. To address this, the first strategy focuses on enhancing psychological capability and promoting reflective motivation of the emergency department staff. The co-design team proposed that the app needs to be included in the emergency department new staff orientation as part of the existing suicide prevention module, as this was not the case. This integration will cover key details about the app, including what it is, how and why it was created and its intended audience. Empirical use cases in emergency departments around the world, specifically related to safety planning and suicide prevention, will need to be included in the module (BCT 5.1: Information about health consequences), along with instructions on how to download, introduce and navigate the app (BCT 4.1: Instruction on how to perform behaviour). The background of the app, which was inspired by an internal staff who pitched the idea of it for his wife who struggled with suicidal thoughts, was also discussed that it needs to be included, along with the app user testimonials (BCT 6.3: Information about others’ approval) and systematic review reports of SPI effectiveness evaluations (BCT 9.1: Credible source). This implementation strategy in a form of a slide deck will be circulated to educators for nursing, social workers and psychiatrists.

‘I think one thing that kind of strikes me is the limited awareness piece… and I feel like there’s a real split among clinicians. There are clinicians who are very familiar with the app and who talk about it with patients… And then on the other hand, you’ve got people who are kind of like, never heard of it… So, I think there’s a really important education piece around the benefits and also just how to use the Hope App for the clinicians in emergency department that has to happen before.’ [Patient, Session 1]

‘More details about the app, which is probably what people need to really understand how it might be helpful. If there are examples of how it’s been used in emergency department settings, that would be good.’ [Clinician, Session 2]

### Education, training and enablement

To address the limited awareness and facilitate greater ‘buy-in’ among the emergency department clinicians, we decided to provide opportunities for brief learning period (BCT: 3.2. Social support (practical)). By utilising built-in emergency department huddle time, education opportunities can be perceived as not burdensome for staff who already have multiple modules to complete on annual basis. The main rationale for this strategy was to navigate the busy nature of the emergency department and make the most of the built-in meetings for emergency department staff. Enablement is a specific intervention function that goes beyond education and training to increase capability, and beyond changing the environment to provide opportunities for behaviour change; therefore, we decided to utilise the existing daily morning huddle in the emergency department.

During the emergency department morning huddle, there will be a dedicated portion of time for all staff to try a grounding technique or box breathing together (BCT: 8.1. Behavioural practice/rehearsal, Self-monitoring of outcomes of behaviour), which are contents of the app (BCT: 5.1 Information about health consequences). Part of this strategy also included a verbal prompt for emergency department staff to try out the Hope App themselves, and explore wellness tools during any downtime in the emergency department to increase familiarity and build habits and skills. However, this was not a reliable opportunity, unlike the morning huddle, as downtime in the emergency department is unpredictable and not always guaranteed. One important consideration of this strategy was related to the actor, who can lead the activity. Clinicians voiced that it would be ideal if this responsibility was assigned to non-emergency department staff, because of the existing commitment, and non-clinician members of the team suggested exploring working with healthcare student volunteers or internal human resources.

‘Team huddle is where every staff member gathers around and discusses various things. So that’s when we can talk about this app.’ [Clinician, Session 2]

‘If I can just say not champions because we have way too many champion needs.’ [Clinician, Session 2]

‘I think like the huddle because I think that’s when you’re getting together every day.’ [Patient, Session 3]

### Coercion and environmental restructuring

The co-design team also prioritised addressing another barrier of the lack of standardised communication and workflows around the SPI. Although this barrier is not specific to the app, it is nonetheless a critical issue that impacts the SPI overall. If left unaddressed, the app will introduce additional socio-technical complexity challenges, further complicating the SPI. To address this, the team suggested making changes in the electronic health record (EHR) documentation form (BCT: 12.1 Restructuring the physical environment). Currently, safety planning is not included in order forms in the EHR, and therefore, completed safety plans get scanned into patients’ record. Also, there are currently no communication methods for the SPI among the emergency department care team. The SPI can be initiated by any emergency department clinician, including psychiatrists, nurses,and social workers. Communication about the SPI – its initiation or completion – takes place verbally. As a team, we were also mindful of the existing documentation burden that clinicians face. Therefore, this strategy will introduce minimal but essential components to be documented. We envision adding checkboxes to indicate the delivery of SPI and additional checkboxes to document its modality (e.g. paper versus app). This implementation strategy enables clinicians’ delivery of SPI accountable and enforces commitment via clinical documentation (BCT: 1.8 Behavioural contract). Moreover, this strategy enables creating a new norm around SPI communication and documentation (BCT 12.2 Restructuring the social environment).

‘Because right now that doesn’t exist and we know that building things in the system and they’re called like forcing functions, right? They help change practice.’ [Clinician, Session 3]

### Persuasion

The above strategy enables data collection for evaluating the impact of the SPI. By enabling data collection and the subsequent evaluation, this implementation strategy aims to improve buy-in for the SPI and the app. One major barrier identified in our previous work was related to the perceived benefit of the SPI in general, as well as the SPI app. Despite the substantial body of evidence supporting the impact of the SPI, disbelief or limited buy-in was identified as a major barrier. Moreover, regarding the app modality specifically, there is limited evidence comparing a digital app to a paper-based approach, because of the limited successful implementation of these digital SPIs. The co-design team was also mindful of challenges related to smart device accessibility among the emergency department patients, which was a major reason for allowing two modalities for the SPI in the emergency department.

The team suggested that once the EHR change was completed, collecting data for evaluation would be straightforward. This implementation strategy focuses on tracking the number of SPIs delivered in the emergency department within the specified time period, the modality of SPI (i.e. app or paper) and associated outcomes of interest, such as critical incidents or time to readmission from the point of discharge. This approach aims to provide feedback on clinicians’ target behaviour itself (BCT 2.2: Feedback on behaviour), as well as information on the results of the target behaviour (BCT 2.7: Feedback on outcome(s) of behaviour). These techniques can help clinicians understand both the actions they need to take and the results those actions yield.

‘I think that’d be good to know but I think the impact of [SPI] would maybe be more important. In terms of sustaining the practice.’ [Clinician, Session 2]

‘I think it can be very effective… there isn’t this information already and I think it can be very moving when you see it actually in numbers.’ [Friend of Patient, Session 3]

### Environmental restructuring and enablement 1

The documentation form addition in the EHR also enables reminder functions to support clinicians’ behaviour change. The team discussed using alerts for the SPI both on the electronic whiteboard connected to the EHR and pop-up alerts within the EHR (BCT 7.1 Prompts/cues, BCT 11.3 Conserving mental resources). However, clinicians reminded the rest of the co-design team about alert fatigue and uncertain effectiveness of EHR alerts. In addition, another restructuring of the digital environment was related to the computer desktops, creating a shortcut icon on a desktop window page for easy access of the app resources (BCT 12.1 Restructuring the physical environment). The main rationale for this strategy and the BCTs were related to conserving mental resources for clinicians, especially during the initial phase of implementation when behaviour change is not familiar. Moreover, recognising that the emergency department is a busy setting with constantly shifting priorities owing to high-acuity levels, the use of cues can help minimise demands on clinicians’ working memory.

‘Whiteboard tool can help, because like a reminder when you see, if flagged, “oh, you have to talk about the Hope App and you have to go through it with the patients.”’ [Clinician, Session 3]

‘We have a similar system for giving the naloxone kits, a reminder to give a naloxone kit at discharge, so the alert, is just there as a prompt.’ [Clinician, Session 3]

## Discussion

Successful and sustained implementation of a new digital tool in healthcare is not easy. This research aimed to co-design tailored implementation strategies to support the integration of an app for SPI. This effort was not about introducing a new evidence-based practice, as SPI was already a standard discharge intervention; however, introducing a new modality (i.e. app) still required tailored and multifaceted efforts to address identified barriers and to leverage facilitators. This study was guided by IKT principles, along with the BCW serving as the primary theoretical foundation for designing the implementation strategies. Successful and sustained implementation of an app and its seamless integration into the existing clinical workflows requires clinicians’ perspective, as they are the ones who will be instructing patients to use the app when delivering the SPI, providing necessary instructions and technical support. Other key knowledge users include patients and families who will be impacted by research findings. Therefore, this study also included emergency department service users and caregivers during the co-design process, ensuring that implementation efforts can also meet or anticipate the future needs of emergency department patients and their caregivers during the SPI process.

Numerous facilitators have been documented in the literature regarding collaborative approaches to research, including establishing equal power balances and avoiding tokenism,^27^ and there are many practical guides on how to achieve this.^28^ Aligned with the current evidence, it was essential to ensure meaningful engagement from all participants to enable mutual relationship in our study. One of the ways to achieve this was to ensure that all partners involved acknowledged and valued what each person brings to the co-design session. It was also important to gauge the team’s level of confidence and comfort throughout all sessions, using the PPEET. Meaningful engagement, however, did not necessarily mean that each participant would generate an equal depth and quantity of insights. Instead, the depth and quantity of insights contributed by each person – though they may appear correlated with how much a particular team member spoke – largely depended on the nature of the implementation problem. Conversations in each session were organic and built upon insights from previous meetings.

Many of the implementation strategies we designed relied on the perspectives of clinicians, particularly regarding affordability and practicality within the emergency department. Since our target behaviour, along with the barriers and facilitators we aimed to address, were specific to the emergency department context, it made sense to draw on clinicians’ insights. However, this reliance led some members of the co-design team to feel that they had limited insights to offer concerning internal feasibility, as indicated in the PPEET surveys: ‘I am enjoying hearing everyone’s perspectives. However, I felt this meeting required more perspectives from internal folks regarding how the capacity of emergency department staff will be compatible with the target behaviour’. In response, the primary author, who facilitated all sessions, reminded the team that their presence was valuable, regardless of how much they contributed verbally. Their presence and feedback were essential for ensuring alignment with the study’s goals and for anticipating the future expectations of emergency department patients and caregivers, regardless of how much they spoke.

One implementation strategy in this study, which includes the introduction of a new documentation form to capture the delivery of SPI, received a unanimous 100% in the APEASE ratings. Despite reported documentation burdens and challenges associated with introducing new charting elements,^29,30^ it was interesting to observe positive reaction from the team. Documentation is a significant part of healthcare, which serves multiple objectives including medical legal, but more importantly, it provides a source of information to shape better care.^31^ However, changing documentation forms or introducing new ones can cause problems, but this is not the case when users are included in the design phase, ensuring that their needs and context are considered.^32^ As such, this new documentation is expected to be well-received by the emergency department team. One other implementation strategy involving peer support workers received the poorest ratings on the APEASE criteria. Although the idea was initially viewed positively for its potential impact on emergency department patients, it generated intense discussion regarding its practicality. In many healthcare organisations, peer support workers play an important role, such as being a role model for recovery.^33^ However, considering the unique nature of the local psychiatric emergency department setting and suicide-related thoughts and behaviours, this idea received significant scrutiny. Currently, there is a peer support worker in the emergency department who comes in once a week and is mainly involved in addiction services, working closely with social workers. The final suggestion we reached by consensus was to further explore whether the emergency department could leverage and delegate tasks related to the SPI app (e.g. ensuring Wi-Fi connectivity, downloading the app and providing instructions) to existing peer support workers, thereby expanding their current responsibilities.

Implementation strategies developed in this study holds promising potential to effectively change clinicians’ behaviour. For example, the EHR documentation addition allows subsequent data collection and evaluation, and this resembles audit and feedback, one of the most popular implementation strategies used to improve quality of healthcare.^34–36^ In our study, incorporating the EHR documentation change and evaluating the impact of SPI are expected to facilitate opportunistic and motivational change of behaviour. Specifically, this implementation strategy encompasses BCT 2.2: Feedback on behaviour and BCT 2.7: Feedback on outcome(s) of behaviour, and the existing literature demonstrates promising effectiveness of these techniques.^34,35^ A recent meta-analysis also demonstrated that education and reminders positively influenced practice change.^36^ Specifically, education, along with audit and feedback, positively influenced clinicians’ attitudes (motivation) and skills (capability),^36^ all of which are implementation determinants identified in the current study context. Moreover, prompts and cues (BCT 7.1 Prompts/cues),^37^ or nudges,^38^ the equivalent of prompts in the Expert Recommendations for Implementing Change compilation,^39^ are widely recognised effective strategies for changing clinicians’ behaviour. Prompts are helpful for emergency department clinicians, as these alleviate mental burden by acting as external memory aids, recognising that emergency departments require continuous reprioritisation of tasks.

### Limitations

The use of behaviour change theory and a collaborative approach in this study is a key strength, addressing a notable gap in the current research in the field of health informatics. Nonetheless, there are limitations that must be acknowledged. The co-design team was relatively small, and the strategies designed are specific to the needs of this psychiatric emergency department in Toronto, Canada, the participants involved and those they represent. Substantiated by our previous qualitative work,^15^ it was also clear that tailored efforts were necessary for this specific context, rather than attempting to generalise findings from a sample to the broader population. Therefore, transferability of our findings and co-design approach cannot be guaranteed in other clinical settings. Necessary adaptations may be required to determine whether these implementation strategies are applicable in other high-acuity environments. Such adaptations can be achieved by conducting a needs assessment of the implementation context and comparing our identified barriers and facilitators to those in the target settings. This process can help identify differences in priorities regarding the most influential barriers to implementation, allowing for reprioritisation of appropriate implementation strategies.

Despite the potential impact of the generated implementation strategies, ongoing discussion is required around whether implementation strategies that target clinicians’ behaviour change has lasting impact on patient outcomes. Clinicians play a critical role in healthcare delivery. Changes in their practice can lead to measurable improvements in patient health, although these outcomes are influenced by multiple factors, ranging from patient engagement to broader healthcare and organisational cultures.^36,40^ Similarly, the current study has an underlying assumption that changes in clinicians’ behaviour can trigger a chain of behaviour changes among patients and families. However, we also acknowledge that patients’ behaviour after emergency department discharge is beyond the control of the emergency department team, like many other chronic conditions that require self-management.

In conclusion, the implementation strategies produced by this study have the potential to address the current gaps in digital technology integration. The BCW and IKT approach used in this study are notable strengths. This have led to the development of 11 implementation strategies that reflect the needs and preferences of clinicians, patients and families. Future testing is required to evaluate the impact of these strategies on the observable and proximal behaviour change among clinicians, along with outcomes related to implementation and sustainability of the app.

## Supporting information

Shin et al. supplementary material 1 Shin et al. supplementary material

Shin et al. supplementary material 2 Shin et al. supplementary material

Shin et al. supplementary material 3 Shin et al. supplementary material

## Data Availability

The data that support the findings of this study are available on request from the corresponding author, H.D.S. The anonymity of the participants must be secured; in the raw data, it is possible to identify the participants, and therefore restrictions will be applied to the availability of these data. Reasonable requests concerning the data can be sent to the corresponding author.

## Supplementary material

The supplementary material is available online at https://doi.org/10.1192/bjo.2025.10824
