# Supplementary material for: Developing theory-informed implementation strategies to embed a suicide safety planning intervention app into a psychiatric emergency department: co-design study using the Behaviour Change Wheel
Source: BJPsych Open. 2025 Sep 12;11(5):e209. doi: 10.1192/bjo.2025.10824 (PMC12451544; doi:10.1192/bjo.2025.10824)
Supplement: Shin et al. supplementary material 1 — Shin et al. supplementary material [file S2056472425108247sup001.docx]

Supplementary File 3: APEASE rating from Session 3 and final recommendations from Session 4

| Intervention functions | Implementation strategies and description of BCTs | Modes of delivery | **Good**: ≥ 80% agreement  **Moderate**: 60–79% agreement  **Poor**: <60% agreement | | | | | | Final recommendations or comments | Relevant quotes |
| --- | --- | --- | --- | --- | --- | --- | --- | --- | --- | --- |
|  |  |  | Affordability | Practicality | Effectiveness | Acceptability | Safety/Side-effects | Equity |  |  |
| 6. Environmental restructuring  +  enablement 1 | - Use of whiteboard in the emergency department to alert clinician (i.e. colored alerts that triggers from the initial emergency department assessment) - EHR pop up alerts - Create shortcut on window page for accessing Hope App information for clinicians - Actors: advance practice nurse, emergency department leadership, IT team - Frequency: one time change | - Digital whiteboard in the emergency department - EHR - Computer desktop icon | Yes: 6  No: 0  Unsure: 1  86% | Yes: 7  No: 0  Unsure: 0  100% | Yes: 6  No: 1  Unsure: 0  86% | Yes: 5  No: 1  Unsure: 1  71% | Yes: 6  No: 0  Unsure: 1  86% | Yes: 7  No: 0  Unsure: 0  100% | - Once EHR documentation is built, this change is relatively easy to make - Worth exploring and need to work closely with advance practice nurse to ensure its feasibility | ‘A couple of thoughts I have. One is alert fatigue, like sometimes if there's a lot of kind of some of the forced functionality ends up just being ignored at times if it's sort of over and over again.’ (Clinician) |
| 7. Environmental restructuring 1 – physical | - Access to the SPI paper pile in the emergency department: consider making the paper pile of safety plan less visible to emergency department clinical staff (i.e. re-location of the paper pile) - Actors: emergency department secretary - Frequency: one time change | Not applicable | Yes: 5  No: 0  Unsure: 2  71% | Yes: 3  No: 1  Unsure: 3  43% | Yes: 3  No: 0  Unsure: 4  43% | Yes: 5  No: 0  Unsure: 2  71% | Yes: 5  No: 0  Unsure: 2  71% | Yes: 4  No: 0  Unsure: 3  57% | - Do not relocate paper - Enable easier access to app instead of making the paper difficult to access - Consider placing QR code on the paper copy (or business card) | ‘If we hide this, to make it more difficult to get, I think it could be problematic.’ (Clinician)  ‘I don't think it's a good idea necessarily for us to make the paper more difficult to access. I really liked [clinician’s] point about just keeping the papers with the rest of the papers where they were, because I think it's really important that there are people who might be intimidated by the use of technology.’ (Patient) |
| 8. Environmental  restructuring 2 – social, inner | - Place pamphlets about the app, including its QR codes, in the emergency department waiting room   - Contents for the pamphlet: What it is, how it was created, who it is for, why it was created, user’s approvals and testimonials for the app - Actors: emergency department secretary - Frequency: one time change | - Print out | Yes: 7  No: 0  Unsure: 0  100% | Yes: 7  No: 0  Unsure: 0  100% | Yes: 7  No: 0  Unsure: 0  100% | Yes: 7  No: 0  Unsure: 0  100% | Yes: 7  No: 0  Unsure: 0  100% | Yes: 6  No: 0  Unsure: 1  86% | - Pamphlets get picked up frequently in the emergency department waiting room - No new suggestions | ‘It's always nice to see like something tangible like that since we had been talking about this in previous sessions, you know, brochures, I think it is always very important to have something tangible with you. It's a great reminder.’ (Patient) |
| 9. Environmental  restructuring 3 – social, outer | - Community promotional posters for the app (e.g. coffee shops around the organisation, subway stations, web) - Target: community - Frequency: undecided | - Poster - Online (i.e. social media platforms like Facebook) | Yes: 4  No: 0  Unsure: 3  57% | Yes: 5  No: 0  Unsure: 2  71% | Yes: 5  No: 0  Unsure: 2  71% | Yes: 7  No: 0  Unsure: 0  100% | Yes: 7  No: 0  Unsure: 0  100% | Yes: 6  No: 0  Unsure: 1  86% | - Finding different avenues (e.g. conferences) for dissemination is a great idea - No new suggestions | ‘These all strike me as being things that, you know, would be nice to do or good to do, in theory, but it all comes down to you know, resources.’ (Patient) |
| 10. Environmental restructuring  +  enablement 2 | - Utilise peer support workers in the emergency department (i.e. Hope App representatives) to introduce this app in the emergency department to patients in the waiting area | - In person | Yes: 2  No: 2  Unsure: 3  29% | Yes: 0  No: 1  Unsure: 6  0% | Yes: 4  No: 1  Unsure: 2  57% | Yes: 4  No: 2  Unsure: 1  57% | Yes: 3  No: 2  Unsure: 2  43% | Yes: 4  No: 2  Unsure: 1  57% | - Explore working with peer support workers who come in once a week (working closely with social workers, part of addictions team) - Consider working with programme assistants - Consider using video instead for this purpose. Video with no audio and subtitle only (in the secure and non-secure waiting area) | ‘I think programme assistants who are assigned to the non-secure waiting area can give a brief explanation and maybe provide a handout regarding the Hope App.’ (Clinician)  ‘I would love to see peer support workers in the emergency department and across the hospital, but again it's like whether those positions are there and if they're able to be created, I think I'd suggest still putting it as a recommendation. ... And who looked at the recommendations, kind of decide if they think it's feasible or not, but I think it's a great recommendation.’ (Friend of patient)  ‘I know her scope is for addictions patients right now, so it'll be more of a limited scope, but if we can try it out, I think that would be a positive thing.’ (Clinician) |
| 11. Incentivisation 2 | - Inform and provide emergency department staff with gift cards to those who provide the app - Whoever provided the app and entered their names in the raffle box can win for a draw - Actors: emergency department team leadership - Frequency: one time activity, collect names for one month | - Gift card | Yes: 6  No: 0  Unsure: 1  86% | Yes: 6  No: 0  Unsure: 1  86% | Yes: 7  No: 0  Unsure: 0  100% | Yes: 7  No: 0  Unsure: 0  100% | Yes: 7  No: 0  Unsure: 0  100% | Yes: 6  No: 0  Unsure: 1  86% | - Consider compensation for all - Some may be indifferent about the raffle - Consider back up plans for financial coverage (e.g. treats) | ‘I think this is a good initiative. I think it adds a bit of an element of fun to the whole thing.’ (Patient)  ‘Some people might feel indifferent to it, but that's fine.’ (Clinician)  ‘I think it's a really simple effective way of incentivizing the use of the Hope App. I would say it's affordable, I'd say it's probably more affordable than marketing and advertisement considering how much that continues to go up in costs depending on where you're doing your advertising. I think [the raffle is] a really simple and effective way of being able to incorporate. I'd be interested in hearing what clinicians here might think about that too, but to me it is what I would have envisioned from the meetings.’ (Patient) |

APEASE, Affordable, Practical, Effective and cost-Effective, Acceptable, Safe and free from unintended negative consequences, and Equitable; BCT, behavioural change technique; EHR, electronic health record; SPI, safety planning intervention.

a. Missing value (*n* = 1).
