# Supplementary material for: Developing theory-informed implementation strategies to embed a suicide safety planning intervention app into a psychiatric emergency department: co-design study using the Behaviour Change Wheel
Source: BJPsych Open. 2025 Sep 12;11(5):e209. doi: 10.1192/bjo.2025.10824 (PMC12451544; doi:10.1192/bjo.2025.10824)
Supplement: Shin et al. supplementary material 2 — Shin et al. supplementary material [file S2056472425108247sup002.docx]

Supplementary File 1. Score (5-point scale) of PPEET items

| **Session #1 (n=7)** | | | |
| --- | --- | --- | --- |
| **PPEET Item** | **Mean** | **SD** | **Range** |
| I have a clear understanding of the purpose of this engagement opportunity.* | 4.8 | 0.4 | 4-5 |
| The supports I need to participate in the co-design session for the Hope App implementation are available (e.g., tech support, orientation) | 4.6 | 0.5 | 4-5 |
| I have enough information to be able to carry out my role. | 4.6 | 0.5 | 4-5 |
| I am able to express my views freely. | 4.4 | 0.8 | 3-5 |
| I feel that my views are heard. | 4.4 | 0.5 | 4-5 |
| A wide range of views on discussion topics is shared.* | 4.7 | 0.5 | 4-5 |
| The individuals participating in this engagement opportunity represent a broad range of perspectives. | 4.4 | 0.8 | 3-5 |
| This co-design session for the Hope App implementation is achieving its stated objectives. | 4.4 | 0.5 | 4-5 |
| I am confident that CAMH takes the feedback provided during this engagement into consideration. | 4.3 | 0.5 | 4-5 |
| I think that the work of co-design session for the Hope App implementation makes a difference to the work of the organization. | 4.3 | 0.5 | 4-5 |
| As a result of my participation in the co-design session, I am better informed about the Hope App and the project's initiatives to support suicide prevention. | 4.4 | 0.5 | 4-5 |
| Overall, I am satisfied with this engagement opportunity. | 4.4 | 0.5 | 4-5 |
| This engagement opportunity is a good use of my time. | 4.4 | 0.5 | 4-5 |
| **Session #2 (n=7)** | | | |
| I have a clear understanding of the purpose of this engagement opportunity. | 4.7 | 0.5 | 4-5 |
| The supports I need to participate in the co-design session for the Hope App implementation are available (e.g., tech support, orientation) | 4.7 | 0.5 | 4-5 |
| I have enough information to be able to carry out my role. | 4.7 | 0.5 | 4-5 |
| I am able to express my views freely. | 4.7 | 0.5 | 4-5 |
| I feel that my views are heard. | 4.6 | 0.5 | 4-5 |
| A wide range of views on discussion topics is shared. | 4.6 | 0.5 | 4-5 |
| The individuals participating in this engagement opportunity represent a broad range of perspectives. | 4.4 | 0.8 | 4-5 |
| This co-design session for the Hope App implementation is achieving its stated objectives. | 4.6 | 0.5 | 4-5 |
| I am confident that CAMH takes the feedback provided during this engagement into consideration. | 4.6 | 0.5 | 4-5 |
| I think that the work of co-design session for the Hope App implementation makes a difference to the work of the organization. | 4.6 | 0.5 | 4-5 |
| As a result of my participation in the co-design session, I am better informed about the Hope App and the project's initiatives to support suicide prevention. | 4.6 | 0.5 | 4-5 |
| Overall, I am satisfied with this engagement opportunity. | 4.6 | 0.5 | 4-5 |
| This engagement opportunity is a good use of my time. | 4.6 | 0.5 | 4-5 |
| **Session #3 (n=7)** |  |  |  |
| I have a clear understanding of the purpose of this engagement opportunity. | 5.0 | 0.0 | 5 |
| The supports I need to participate in the co-design session for the Hope App implementation are available (e.g., tech support, orientation) | 5.0 | 0.0 | 5 |
| I have enough information to be able to carry out my role. | 5.0 | 0.0 | 5 |
| I am able to express my views freely. | 4.7 | 0.5 | 4-5 |
| I feel that my views are heard. | 4.6 | 0.8 | 3-5 |
| A wide range of views on discussion topics is shared. | 4.9 | 0.4 | 4-5 |
| The individuals participating in this engagement opportunity represent a broad range of perspectives. | 4.9 | 0.4 | 4-5 |
| This co-design session for the Hope App implementation is achieving its stated objectives. | 4.9 | 0.4 | 4-5 |
| I am confident that CAMH takes the feedback provided during this engagement into consideration. | 4.6 | 0.8 | 3-5 |
| I think that the work of co-design session for the Hope App implementation makes a difference to the work of the organization. | 4.9 | 0.4 | 4-5 |
| As a result of my participation in the co-design session, I am better informed about the Hope App and the project's initiatives to support suicide prevention.* | 4.8 | 0.4 | 4-5 |
| Overall, I am satisfied with this engagement opportunity.* | 4.8 | 0.4 | 4-5 |
| This engagement opportunity is a good use of my time. | 4.9 | 0.4 | 4-5 |
| **Session #4 (n=11)** | | | |
| I have a clear understanding of the purpose of this engagement opportunity. | 4.8 | 0.4 | 4-5 |
| The supports I need to participate in the co-design session for the Hope App implementation are available (e.g., tech support, orientation) | 4.7 | 0.6 | 3-5 |
| I have enough information to be able to carry out my role. | 4.5 | 0.7 | 3-5 |
| I am able to express my views freely. | 4.8 | 0.4 | 4-5 |
| I feel that my views are heard. | 4.8 | 0.4 | 4-5 |
| A wide range of views on discussion topics is shared. | 4.7 | 0.6 | 3-5 |
| The individuals participating in this engagement opportunity represent a broad range of perspectives. | 4.8 | 0.4 | 4-5 |
| This co-design session for the Hope App implementation is achieving its stated objectives. | 4.8 | 0.4 | 4-5 |
| I am confident that CAMH takes the feedback provided during this engagement into consideration. | 4.7 | 0.5 | 4-5 |
| I think that the work of co-design session for the Hope App implementation makes a difference to the work of the organization. | 4.6 | 0.5 | 4-5 |
| As a result of my participation in the co-design session, I am better informed about the Hope App and the project's initiatives to support suicide prevention. | 4.6 | 0.5 | 4-5 |
| Overall, I am satisfied with this engagement opportunity. | 4.8 | 0.4 | 4-5 |
| This engagement opportunity is a good use of my time. | 4.7 | 0.5 | 4-5 |
| *Missing value n=1 | | | |
