# Supplementary material for: Developing theory-informed implementation strategies to embed a suicide safety planning intervention app into a psychiatric emergency department: co-design study using the Behaviour Change Wheel
Source: BJPsych Open. 2025 Sep 12;11(5):e209. doi: 10.1192/bjo.2025.10824 (PMC12451544; doi:10.1192/bjo.2025.10824)
Supplement: Shin et al. supplementary material 3 — Shin et al. supplementary material [file S2056472425108247sup003.docx]

Supplementary File 2. Implementation strategies made up of BCTs and Justifications

| Intervention functions | Brief description of the implementation strategy | Behaviour Change Techniques (BCTs) | Justification (Target of BCTs) | COM-B target(s) of change |
| --- | --- | --- | --- | --- |
| 1. Education  +  Training  +  Persuasion | Education and training module for the ED staff | 5.1. Information about health consequences  4.1. Instruction on how to perform behaviour  9.1. Credible source  6.3. Information about others’ approval | - Limited awareness and use of the Hope App - Uncertain benefits (i.e., health consequences) of safety planning beyond discharge - Address perceived benefit and disadvantages of the app | C, M |
| 2. Incentivization 1 | Certificate of completion for the training module described above | 10.1. Material incentive (behaviour)  10.2. Material reward (behaviour) | - Buy-in of the app - Optimism towards the app implementation - Reinforcement | M |
| 3. Education  +  Training  +  Enablement | Providing opportunity during the ED shift for education and training | 5.1 Information about health consequences  3.2. Social support (practical)  8.1. Behavioural practice/rehearsal | - Limited awareness of the app - Social influence among the ED team - Leverage team culture in the ED - Address perceived benefit and disadvantages of the Hope App (i.e., buy-in and health consequences of SPI impact) - Provide opportunity within the busy ED to improve familiarity of the app | C, O, M |
| 4. Coercion + Environmental restructuring | Electronic health record documentation form | 1.8. Behavioural contract  12.1 Restructuring the physical environment  12.2 Restructuring the social environment | - Professional responsibility and communication for SPI | O, M |
| 5. Persuasion | Evaluation of SPI | 2.2. Feedback on behaviour  2.7. Feedback on outcome(s) of behaviour | - Uncertain benefits of safety planning beyond discharge - Perceived benefit and disadvantages of the Hope App | M |
| 6. Environmental restructuring  +  Enablement 1 | Use of technology-enabled alerts and reminders | 7.1. Prompts/cues  12.1. Restructuring the physical environment  11.3. Conserving mental resources | - Busy ED setting and being understaffed, need for prioritizing multiple tasks - Memory, attention and decision making: Remembering the app especially in the beginning phase of change - Need for standard communication for SPI in the ED - Accessible, readily available resources for the ED staff - Professional responsibility for SPI in the ED | C, O |
| 7. Environmental restructuring 1 - Physical | Access to the SPI paper pile in the ED | 12.1. Restructuring the physical environment | - Behavioural regulation and habit reversal: Opting for the quickest way of handing out paper, hence need to break the habit | C, O |
| 8. Environmental  restructuring 2 – Social, Inner | Pamphlets in the ED waiting room | 5.1. Information about health consequences  12.2. Restructuring the social environment  12.5. Adding objects to the environment | - Limited awareness of the Hope App amongst the broader community - Promoting patients’ awareness of the app - Interested patients can download the app while waiting in the ED and in doing so, this can save time during discharge; this can help support busy ED setting being understaffed, which requires staff to prioritizing multiple tasks | C, O |
| 9. Environmental  restructuring 3 – Social, Outer | Community promotional activities | 12.2. Restructuring the social environment | - Limited awareness of the Hope App amongst the broader community - Future patients’ familiarity and willingness to use the app | C, O |
| 10. Environmental restructuring  +  Enablement 2 | Peer support workers in the ED | 12.2. Restructuring the social environment  3.2. Social support (practical) | - Busy ED setting and being understaffed, need for prioritizing multiple tasks - Patients’ acceptability of the app can be improved if introduced by peers, who share similar experiences | O |
| 11. Incentivization 2 | Raffle draws for ED staff | 10.1. Material incentive (behaviour)  10.2. Material reward (behaviour) | - Limited awareness of the Hope App amongst the ED staff - Perceived benefit and disadvantages of the Hope App - Uncertain benefits of safety planning beyond discharge - Reinforcement | M |
